# Hyperosmolality in CHO cell culture: effects on the proteome

**DOI:** 10.1007/s00253-022-11861-x

**Published:** 2022-03-21

**Authors:** Nadiya Romanova, Louise Schelletter, Raimund Hoffrogge, Thomas Noll

**Affiliations:** Cell Culture Technology, Technical Faculty, Bielefeld University, Universitätsstraße 25, 33615 Bielefeld, Germany

**Keywords:** CHO, Fed-batch, Hyperosmolality, Cell size, LFQ proteomics

## Abstract

Chinese hamster ovary (CHO) cells are the most commonly used host cell lines for therapeutic protein production. Exposure of these cells to highly concentrated feed solution during fed-batch cultivation can lead to a non-physiological increase in osmolality (> 300 mOsm/kg) that affects cell physiology, morphology, and proteome. As addressed in previous studies (and indeed, as recently addressed in our research), hyperosmolalities of up to 545 mOsm/kg force cells to abort proliferation and gradually increase their volume—almost tripling it. At the same time, CHO cells also show a significant hyperosmolality-dependent increase in mitochondrial activity. To gain deeper insight into the molecular mechanisms that are involved in these processes, as detailed in this paper, we performed a comparative quantitative label-free proteome study of hyperosmolality-exposed CHO cells compared with control cells. Our analysis revealed differentially expressed key proteins that mediate mitochondrial activation, oxidative stress amelioration, and cell cycle progression. Our studies also demonstrate a previously unknown effect: the strong regulation of proteins can alter both cell membrane stiffness and permeability. For example, we observed that three types of septins (filamentous proteins that form diffusion barriers in the cell) became strongly up-regulated in response to hyperosmolality in the experimental setup. Overall, these new observations correlate well with recent CHO-based fluxome and transcriptome studies, and reveal additional unknown proteins involved in the response to hyperosmotic pressure by over-concentrated feed in mammalian cells.

**Key points**

• *First-time comparative proteome analysis of CHO cells exposed to over-concentrated feed.*

• *Discovery of membrane barrier-forming proteins up-regulation under hyperosmolalit*y.

• *Description of mitochondrial and protein chaperones activation in treated cells*.

**Supplementary Information:**

The online version contains supplementary material available at 10.1007/s00253-022-11861-x.

## Introduction

The biopharmaceutical and biotechnological importance of CHO cells is indisputable. Their robust growth in suspension, their relatively easy genetic manipulation, and their capacity for protein folding and post-translational modification make them the preferred expression system across a wide variety of biopharmaceutical products (Sharker and Rahman 2020).

To achieve maximum cell density and product titer, CHO cells are typically cultivated in a fed-batch mode, with either continuous or bolus feed addition. The feed used is a highly concentrated nutrient solution, which may cause an increase in osmolality within the culture. Exposure of CHO cells to hyperosmolality can lead to substantial adaptation effects (such as decreased proliferation), but also to an unexpected increase in cell size. This effect has been widely observed and reported in the relevant literature (Kiehl et al. 2011; Pan et al. 2017; Takagi et al. 2000). In previous research, we have also shown that a step-wise increase in osmolality during fed-batch cultivation not only terminates cell proliferation and leads to the cell size increase, but also causes significant hyperosmolality-dependent mitochondrial activation without concomitant apoptosis induction (Romanova et al. 2021).

The most common reagent in hyperosmolar studies is NaCl (Han et al. 2010; Kiehl et al. 2011; Lee et al. 2017; Lin et al. 1999). It is not inert with respect to cells—in addition to osmotic effects, it can also cause specific metabolic changes (Dmitrieva et al. 2011; Kultz and Chakravarty 2001) that should ideally be avoided. Indeed, even some substances that were previously considered to be inert with respect to cells (such as sucrose or maltose) have recently been shown to actually be consumed and processed (Leong et al. 2018). There are also substances whose status is not yet confirmed with certainty: for example, it remains presently unknown whether mannitol or L-glucose used as an osmotic control may have as-yet unknown effects on the cell. Moreover, these substances are absent in typical industrially used feed or growth medium. Surveying all of these different considerations, we decided to focus our attention on the modulation of osmolality in culture by adding an industrially relevant, oversupplemented feed. Here, D-glucose serves as the main component to control osmolality. A previous study by Madonna et al. (2016) compared the effects of incubation with high-D-glucose, high-mannitol, high-L-glucose, and high-NaCl media on human endothelial cells. In that study, the authors found a comparable increase in osmolality-specific protein expression—suggesting that a high concentration of all of those solutes may induce similar molecular responses.

The cellular response to hyperosmolality has been a focus of recent CHO-based fluxome (Pan et al. 2017) and transcriptome (Bedoya-López et al. 2016; Pan et al. 2019; Shen et al. 2010) studies. Having said that, the most recent proteome study dates all the way back to 2003 (Lee et al. 2003)—when a total of twenty-three different proteins were identified by two-dimensional gel electrophoresis and mass spectrometry identification, but only three of them appeared to show significant expression levels dependent on hyperosmolality. Since that time, rapid technological advances in mass spectrometry equipment and data evaluation/mining capabilities as a basis for large-scale CHO-specific proteomic analyses (Baycin-Hizal et al. 2012; Geiger et al. 2012; Heffner et al. 2020; Schelletter et al. 2019) have dramatically changed the depth, precision, and propensity for protein identification of proteome studies. Harnessing those technology advancements, in this paper, we describe the results of much more recent analysis of comparative proteomes based on nano-scale liquid chromatography-electrospray ionization tandem mass spectrometry (nLC-ESI–MS/MS) measurements that reveal new adaptation effects to hyperosmolality in CHO cells at the proteome level.

## Materials and methods

### Experimental setup

A detailed description of cell culture maintenance and experimental setup for fed-batch cultivation has been detailed at length in previous studies (Romanova et al. 2021). In brief, however, in our work covered in this paper, we studied the effects of high-osmolality feeding on suspension-adapted, antibody-producing CHO DP-12 clone#1934 (ATCC CRL-12445) cells.

For this purpose, we cultivated the cells in shaking flasks with at least three biological replicates per condition. For fed-batch cultivation, cells were seeded at a starting viable cell density (VCD) of 3 × 10^5^ cells/ml in 40 ml growth medium TCX6D, supplemented with 6 mM Gln (Xell AG, Bielefeld, Germany). Six milliliters of either highly supplemented and industrially relevant feed solution (“feed” condition) or supplemented medium (“control” condition) was thereafter added four times each on days 3, 4, 5, and 6. We used CHO Basic Feed (Xell AG) supplemented with 404 mM D-glucose, 70 mM glutamine, and 27 mM asparagine (osmolality of pure supplemented feed: 830 mOsm/kg). The supplemented feed that was administered contained a subset of components already present in the basal growth medium, and did not introduce any new components to the feed condition. This feeding strategy resulted in a step-wise increase in osmolality in feed up to 545 mOsm/kg, with no observed change in osmolality in “control.” We observed that cell proliferation in feed was completely inhibited after the second feeding—accompanied by a concomitant increase in cell size. In our experimental setup, D-glucose was identified as the main cause of increasing osmolality within the fed-batch. To exclude the toxic influence of glycolysis products, lactate concentration was also measured daily to confirm that its accumulation remained at a relatively low level (not exceeding 25 mMol/l) that is known to be well-tolerated by cells (Hartley et al. 2018). Further information on the effects on growth, cell size, and productivity is presented by Romanova et al. (Romanova et al. 2021).

### Cell lysis and protein digestion

**Fig. 1:**
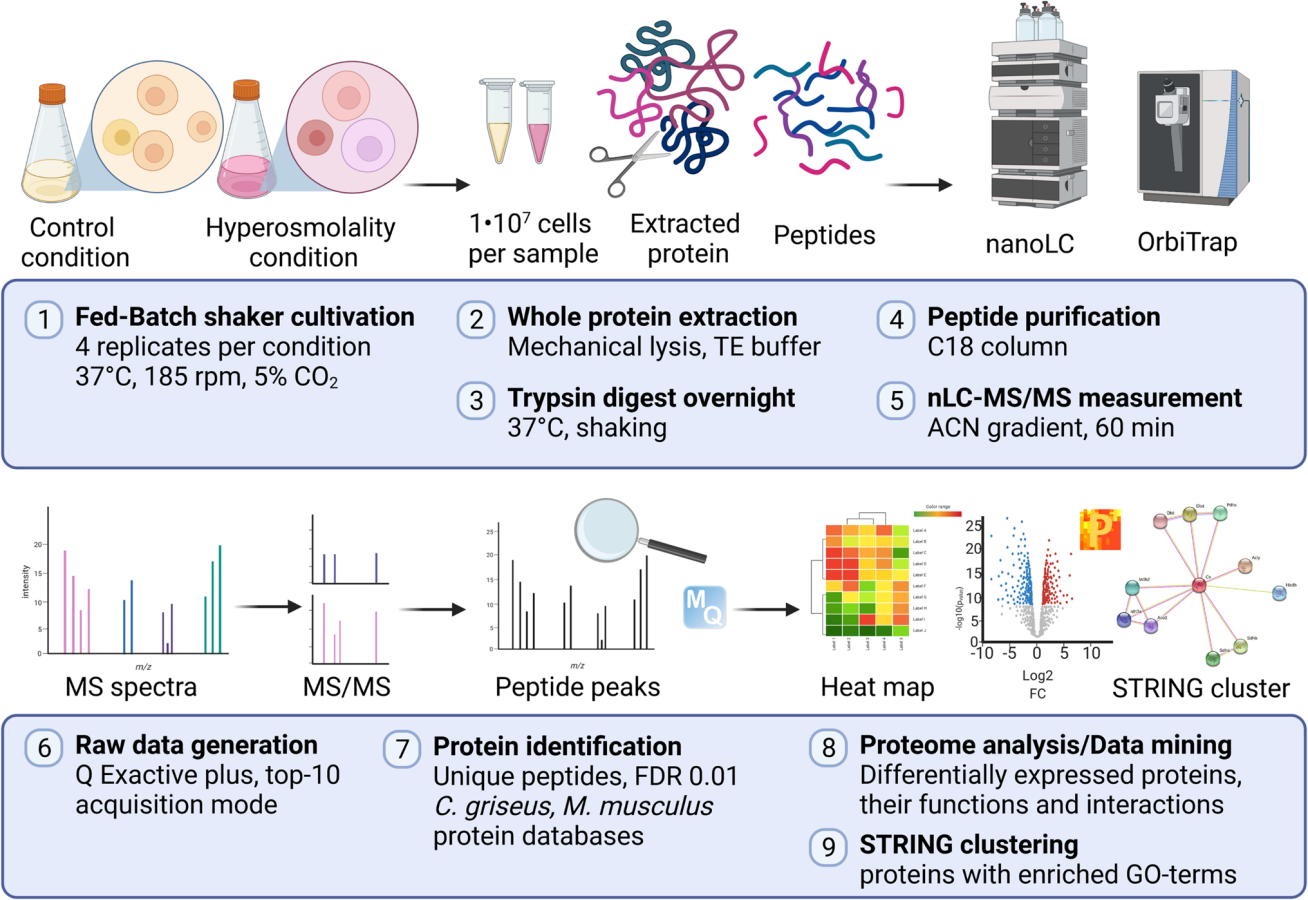
Workflow of LFQ proteomics used in this work. Created with BioRender.com (https://biorender.com/)

The workflow of differential protein expression analysis via label-free quantification mass spectrometry (LFQ-MS) is presented in Fig. 1. For LFQ-based comparison of proteomes, 1 × 10^7^ cells of four biological replicates for each of the two conditions (“feed” and “control”) were harvested on days 2, 6, and 8. The cells were centrifuged at 200 × *g* for 5 min and then washed once in PBS, and cell pellets were thereafter stored at − 80 °C until further analysis.

Cells were resuspended in 300 µl of 50 mM Tris-buffer pH 8.0 and transferred into a new reaction tube containing approximately 150 µl of 0.1-mm glass grinding beads (Carl Roth GmbH, Karlsruhe, Germany). All samples were processed in batch and then randomized early in the workflow, in order to minimize any possible biases. Cells were mechanically ruptured by vortexing 4 × 30 s and also cooled on ice after each cycle. The supernatant was collected after 30-min centrifugation (17,000 × g, at 4 °C), and the protein concentration was quantified via a bicinchoninic acid assay (BCA). Twenty micrograms of whole-cell protein was precipitated with nine parts of ice-cold acetone overnight at − 20 °C. Afterwards, the samples were centrifuged for 30 min at 17,000 × g; acetone was discarded; and any residual acetone was allowed to evaporate. Samples were then rehydrated in 50 mM Tris–HCl buffer, pH 8.0. Next, reduction (7 mM dithiothreitol (DTT), 30 min, 60 °C, shaking 300 rpm), alkylation of cysteine residues (20 mM iodoacetamide (IAA), 30 min, room temperature, dark), and quenching by adding 14 mM DTT (40 min, room temperature, 300 rpm) were all sequentially performed. In-solution trypsin/Lys-C (mix of Trypsin Gold and recombinant Lys-C Promega, Mannheim, Germany) digestion of 20 μg protein was performed overnight in a 1:10 dilution of 50 mM Tris–HCl buffer (pH 8.0), according to the manufacturer’s instructions (25:1 protein:protease w/w ratio). To remove any remaining potential impurities and detergents, peptides were purified via C18 SepPak C18 vac 1 cc (Waters, Milford, MA) columns. In a nutshell, the column material has a strongly hydrophobic surface that was able to retain even mildly hydrophobic substances. Finally, elution was performed twice by 200 µl of 80% acetonitrile (ACN) in LC–MS grade water (Merck, Darmstadt, Germany).

### Western blot

**Fig. 2:**
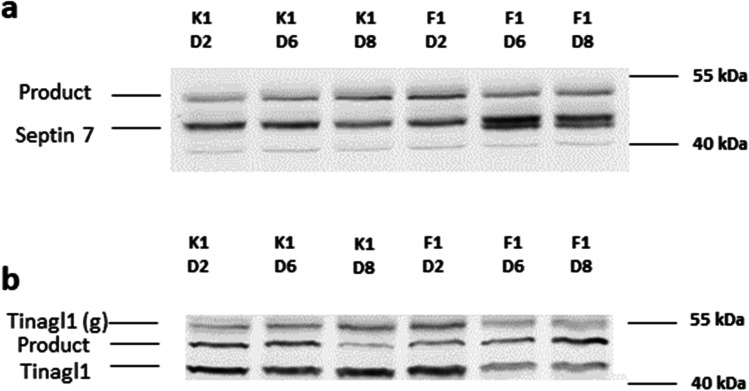
Western blot analysis of oversupplemented feed-exposed (F) and control (K) whole protein lysates of the fed-batch cultivation of CHO-DP12 sampled on days 2, 6, and 8. **a** Antibody against septin 7, detecting a band at 50 kDa next to product. **b** Antibody against Tinagl1, detecting a specific band at about 52 kDa, at expected Tinagl1 size (glycosylated) and an additional band at 44 kDa (verified by HEK-cell line lysate), probably unglycosylated

Western blots were performed on the same samples used for MS analysis (Fig. 2). Proteins were extracted from 1 × 10^7^ cells, and quantified as described above. Twenty micrograms of protein was separated via either a 12.5% (glutathione peroxidase Gpx1 and septin 7 antibodies) or a 10% (tubulointerstitial nephritis antigen-like, Tinagl1) SDS-PAGE in an electrophoresis chamber (Bio-Rad Laboratories, Feldkirchen, Germany). Subsequently, the separated proteins were transferred from the gels to an immune-blot fluorescence polyvinylidene difluoride (PVDF) membrane (Bio-Rad Laboratories, Feldkirchen, Germany), using a semi-dry environment (XCell II™ Blot Module, Thermo Fisher Scientific, Dreieich, Germany) in a Bis–Tris/Bicine buffer that contained 10% ethanol. The membranes were then blocked for 1 h at room temperature using 5% milk powder in Tris-buffered saline with 0.1% Tween 20 (TBS-T). The following primary antibodies were used in specified dilutions: Gpx1 Polyclonal Antibody # PA5-88,073 1:12 000 and Tinagl1 Polyclonal Antibody # PA5-89,001 (all Thermo Fisher Scientific, Dreieich, Germany) 1:1000 both in 5% milk powder in TBS-T; septin 7 Polyclonal Antibody # PA5-56,181 1:1000 in 2.5% milk powder in TBS-T. The antibodies were thereafter incubated overnight at 4 °C. Secondary anti-rabbit Cy3-labeled antibody # 111–165-003 (Dianova, Hamburg, Germany) was used in 1:1000 dilution in TBS-T containing 2.5% milk powder. Enhanced chemiluminescence (ECL)-anti-rabbit antibody # 7074 (Cell Signaling Technology, Inc., Frankfurt am Main, Germany) was used in 1:4000 dilution in 1% milk powder in TBS-T. Secondary antibodies were incubated for 2 h in the dark at room temperature. The ECL antibody was also thereafter incubated overnight at 4 °C. The detection was performed at 570 nm emission with an Ettan DIGE Imager (GE Healthcare, Solingen, Germany) or Fusion Fx7 CCD-Camera (Vilber Lourmat, Eberhardzell, Germany), respectively.

### nLC-ESI–MS/MS

Purified peptide mixtures of 20 μg digested whole-cell lysate were resolubilized in 11 μl of LC–MS grade water with 0.1% trifluoroacetic acid (TFA) and 2.5% acetonitrile (ACN). Peptide concentrations (standard deviation (sd) < 10%, absorbance at 205 nm) were measured by NanoDrop One (Thermo Fisher Scientific, Dreieich, Germany) using 1 µl of the sample volume, which facilitated the injection of normalized peptide amounts for nLC-MS/MS measurements. For reversed-phase peptide separation, an UltiMate 3000 RSLC nanoLC Dionex system (Thermo Fisher Scientific, Dreieich, Germany) with Acclaim PepMap™ 100 C18 pre-column cartridge (300 µm I.D. × 5 µm, Thermo Fisher Scientific) and a 25 cm Acclaim PepMap™ 100 C18 separation column (2 µm, 75 µm I.D., Thermo Fisher Scientific, Dreieich, Germany) with an effective gradient of 1–50% solvent B (80% ACN, 1% TFA) at a flow rate of 300 nl/min was run for 60 min. Online ESI-Orbitrap mass spectrometry measurements were carried out using a Q Exactive Plus instrument (Thermo Fisher Scientific, Dreieich, Germany) in data-dependent top ten acquisition mode, with a minimum automatic gain control (AGC) value of 1e3, and a dynamic exclusion time of 20 s. Precursor ions were acquired in MS mode with a resolution of 70,000, an AGC target of 3e6, and 80 ms maximum IT. Fragment ions were scanned with a resolution of 17,500, an AGC target of 2e5, and an intensity threshold of 120 ms, and peptides were selected in a 1.6 mass-to-charge (m/z) isolation window for fragmentation with normalized collision energy (CE) of 28.

### nLC-ESI–MS/MS data analysis

The complete LFQ-protein quantification and identification were performed in MaxQuant version 1.6.10.43 (Tyanova et al. 2016). Peptide quantification was performed only for unique peptides. Identification was performed using both *Cricetulus griseus* and *Mus musculus* (UniProtKB, download on 08.07.2020, 56,327 entries for *C.* *griseus* and 69,504 for *M.* *musculus*) databases. The taxonomy of each hit is included in the Supporting Information, Table S1. The following modifications were set: cysteine carbamidomethylation (fixed modification), oxidation of methionine, and N-terminal acetylation (variable modifications). The maximum number of missed cleavages for tryptic digest was set to two, and a false discovery rate (FDR) of 0.01 was selected for protein and peptide identification. The resulting data was subsequently processed in Perseus version 1.6.14.0 (Tyanova et al. 2016). The mass spectrometry proteomics data was deposited to the ProteomeXchange Consortium via the PRIDE (Perez-Riverol et al. 2019) partner repository with the dataset identifier PXD027443.

The evaluation workflow was set according to guidelines that have been previously published by Tyanova and Cox (2018), which cover the following steps: excluding in “proteingroups.txt” proteins only identified by site, potential contaminants, and any proteins matching reverse database. The intensities were log2 transformed and filtered for valid values (found in at least three samples). The entries were complemented by gene annotations from Gene Ontology Cellular Component (GOCC), Reactome, Gene Set Enrichment Analysis (GSEA), Kyoto Encyclopedia of Genes and Genomes (KEGG) (Kanehisa et al. 2016), CORUM Molecular Complexes Database (Ruepp et al. 2007), Mouse Genome Identifier (MGI), Evolutionary genealogy of genes: Non-supervised Orthologous Groups (eggNOG) (Huerta-Cepas et al. 2018), SMART/PFAM (Letunic and Bork 2017), and InterPro (Finn et al. 2016).

Categorical column annotation included three sampling points (day 2, day 6, and day 8). Each group included four biological replicates per condition (i.e., “feed” and “control”). Categorical columns were subjected to a two-sided two-sample *t* test, which was used to determine if the mean LFQ-intensity values of two samples or groups were significantly different from each other. For the two-sample *t* test, permutation-based FDR was set to < 0.05, the probability value *p* < 0.02 was considered to be the threshold for significance, and S0 was set to 0. Differentially expressed proteins between C and F conditions on days 6 and 8 were visualized via hierarchical clustering as a heat map. After applying the two-sample *t* tests (“feed” vs. “control” on day 6 and day 8), we also used a Fisher exact test to identify any significantly enriched terms in gene annotations of differentially expressed genes. All graphics represented here were directly exported from the Perseus environment.

The STRING (Search Tool for the Retrieval of Interacting Genes/Proteins) was used for critical assessment and integration of protein–protein interactions (http://string-db.org/). The interactions were drawn from direct experimental evidence and are predicted based on similarities known for other organisms (Chan 2006). By using STRING, the 41 proteins resulting from Fisher exact test based on significantly regulated proteins between “feed” and “control” for day 8 (see Table 1, except for nidogen 1.2, as only one isoform is listed in the database) were mapped and a network image was created (Fig. 4).

**Table 1:** Genes with significant enriched molecular annotations based on differentially expressed proteins (*p* < 0.01) between “feed” and “control” condition found on proteome level during the fed-batch cultivation of CHO DP-12 cells on day 8. The letter *m* stands for “mitochondrial”

	Gene name	Protein name	UniProt AC	Mean log2 fold change day 8 “feed”/ “control”	Biological function
I. Genes in connection with extracellular matrix/membrane					
1	*Col6a1*	Collagen alpha-1(VI) chain	G3H8Y5	− 2.82	Part of extracellular matrix
2	*Lama5*	Laminin subunit alpha-5	G3HGW6	− 1.57	Integrin binding, basement membrane
3	*Tinagl1*	Tubulointerstitial nephritis antigen-like	G3H1W4	− 2.64	Laminin binding, cys-type peptidase
4	*I79_018113*	Nidogen 1.1	G3I3U5	− 1.13	Basement membrane protein
5	*I79_015301*	Nidogen 1.2	G3HWE4	− 0.91	Cell matrix adhesion
6	*Hspg2*	Heparan sulfate core protein (preliminary)	A0A3L7I8L8	− 1.99	Basement membrane protein
7	*Sparc*	Kazal-like domain-containing protein	G3H584	− 1.69	Collagen binding, calcium ion binding
8	*App*	Amyloid-beta A4 protein	G3HMG4	− 1.88	Integral membrane component
II. Genes involved in stress response					
1	*Hsp90b1*	HSP90, beta	Q91V38	0.71	Unfolded protein binding
2	*Gpx4*	Glutathione peroxidase-4	G3HF60	1.04	Glutathione peroxidase activity
3	*Clu*	Clusterin	G3HNJ3	0.80	Protein folding chaperone
4	*Hsph1*	Heat shock 105 kDa protein	G3GWF4	0.51	Prevents protein aggregation
5	*Psmb5*	Proteasome subunit beta type-5	G3HRD9	0.77	Response to oxidative stress
6	*Hspa9*	Stress-70 protein, mitochondrial	G3HEZ0	0.83	Unfolded protein binding
7	*Pxdn*	Peroxidasin-like protein	G3HBI1	− 0.67	Response to oxidative stress
8	*Ciapin1*	Anamorsin	G3HIL4	− 0.91	Anti-apoptotic effector
9	*Anxa1*	Annexin A1	G3I5L3	− 0.72	Inflammatory response
10	*Glg1*	Golgi apparatus protein 1	G3I369	− 1.95	Neg. regulation of protein processing
III. Genes involved in mitochondrial regulation and function					
1	*Acadvl*	VLC-specific acyl-CoA dehydrogenase (m)	G3GYA2	1.19	Fatty acid β-oxidation
2	*Ethe1*	Persulfide dioxygenase ETHE1 (m)	A0A061HTS8	1.01	Suppress p53-induced apoptosis
3	*Hmgcl*	Hydroxymethylglutaryl-CoA lyase (m)	G3HMV6	0.77	Lipid metabolic process
4	*Mdh2*	Malate dehydrohenase (m)	G3HA23	0.89	Carbohydrate metabolic activity
5	*Grpel1*	GrpE protein homolog 1 (m)	G3GWC4	0.98	Protein folding, mitochondrial import
6	*Hadha*	Trifunctional enzyme subunit alpha (m)	G3GXQ3	0.79	Fatty acid β-oxidation
7	*Hsd17b10*	3-hydroxyacyl-CoA dehydrogenase	G3H7U0	0.74	Fatty acid β-oxidation
8	*Pdha1*	Pyruvate dehydrogenase E1, subunit α	G3H5K6	0.88	Pyruvate oxidation
9	*Fdxr*	NADPH:adrenodoxin oxidoreductase (m)	G3GTG7	0.52	Cholesterol metabolism
10	*Ivd*	Isovaleryl-CoA dehydrogenase (m)	G3ICJ8	1.18	Leucine catabolism
11	*Dld*	Dihydrolipoyl dehydrogenase (m)	G3H8L2	0.94	Mitochondrial e- transport
12	*Cpt2*	Carnitine O-palmitoyltransferase 2 (m)	G3GTN3	0.62	Fatty acid metabolism
13	*Hagh*	Hydroxyacylglutathione hydrolase (m)	G3HBP3	0.98	Glutathione metabolism
14	*Shmt2*	Serine hydroxymethyltransferase (m)	G3HW36	0.65	Tetrahydrofolate interconversion
15	*Ssbp1*	Single-stranded DNA-binding protein (m)	G3HGL0	1.51	Mitochondrial DNA replication
16	*Cs*	Citrate synthase (m)	G3HRP3	0.82	Oxidative metabolism, tricarboxylic acid (TCA) cycle
17	*Sdhaf2*	Succinate dehydrogenase assembly f.2 (m)	G3IER1	0.46	Chaperone
18	*Tufm*	Elongation factor Tu (m)	G3GX09	0.90	Mitochondrial elongation translation
IV. Genes involved in cell cycle progression					
1	*Snx9*	Sorting nexin-9	G3HFW9	− 0.82	Mitotic cytokinesis
2	*Ddx3x*	ATP-dependent RNA helicase	G3GSH5	− 0.62	Promotes G1/S-phase cell cycle transition
3	*Stat3*	Signal transducer and activator of transcription 3	G3HLW9	− 1.49	Regulation of cell cycle and transcription
V. Non-mitochondrial metabolic processes					
1	*Nucb2*	Nucleobindin	G3IF52	0.59	Calcium-level maintenance
2	*Eea1*	Early endosome antigen	G3I600	− 0.42	Endosomal trafficking
3	*Phgdh*	D-3-phosphoglycerate dehydrogenase	G3HP75	− 0.98	L-serine biosynthesis

## Results

### Protein identification and quantification

**Fig. 3:**
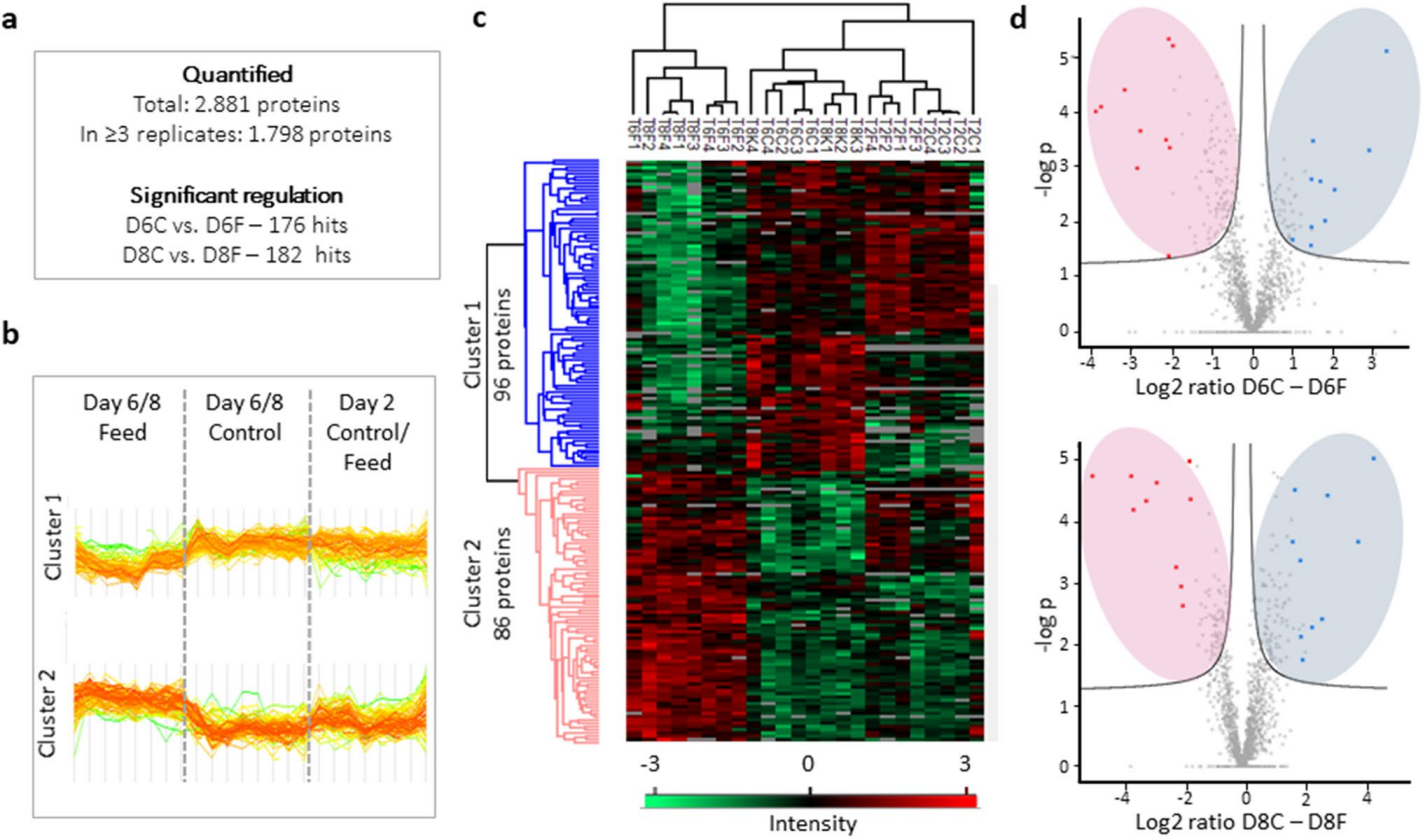
Proteome analysis data across three cultivation time points for the fed-batch cultivation of CHO DP-12 cells exposed to high osmolality (“feed,” F) or without osmotic change (“control,” C). **a** Numbers of quantified and significantly regulated (permutation-based FDR < 0.05) proteins between F and C found in CHO proteomes; **b** profile plots of the log2-ratios F vs. C of the two clusters based on heat map c): cluster 1, proteins with significantly decreased expression on day six and eight in the “feed” condition; 2, proteins with a significantly increased expression on day six and eight in the “feed” condition. **c** Hierarchical clustering of significantly regulated proteins across three cultivation time points for the fed-batch cultivation of CHO DP-12 cells. High and low expression is shown in red and green, respectively (“T” is an abbreviation for “day” (Tag), “C” indicates “control,” and “F” indicates “feed” condition. Number after the letter indicates biological replicate). **d** Volcano plots of fold change (LFQ-intensity) in four biological replicates for significantly regulated proteins on day six and day eight in F and C. The plot is represented as a function of statistical significance (*t* test *p* ≤ 0.01) between “control” and “feed” condition isolates. The Y-axis indicates *p* value (− log 10). The X-axis shows the protein ratio (log2 change) in C vs. F conditions. Proteins significantly up-regulated in the feed are highlighted with pink ovals, proteins significantly down-regulated with light blue ones. The top ten proteins (up-regulated in feed) are marked with red dots; the top ten proteins down-regulated in “feed”—blue dots. Proteins with no statistically significant expression differences between the two conditions are shown in gray under the significance cut-off curve. The S0 parameter was set to 0

For LFQ-MS intracellular proteome analysis, samples were harvested from four biological replicates at three time points: day 2 (i.e., the beginning of the exponential phase and a reference point for both “control” and “feed” conditions), day 6 (i.e., the exponential phase), and day 8 (i.e., the stationary phase and the onset of glucose-limitation in the “control” fed-batch). After sample preparation and tryptic digest, measurements were performed in a randomized order in one batch of nanoLC-ESI–MS acquisitions. Based on 16,660 unique peptides, a total of 2881 non-redundant proteins were identified for CHO-DP12 cells in our experimental setup. A complete list of the identified proteins is available as electronic supplementary material (Supplementary Information, Table S2). After we filtered the results down to only those proteins which occurred in at least three samples, a total of 1798 such proteins were quantified (Fig. 3a; Tables S1 and S2). The coverage accords well with results that have been reported in other recent LFQ-MS CHO proteome studies (Kaushik et al. 2020; Schelletter et al. 2019).

### Differentially expressed proteins: “feed” vs. “control”

The biological replicates for each condition were grouped categorically for further processing. Two-sample *t* tests (permutation-based FDR < 0.05 on day 6 “feed” vs. “control” and day 8 “feed” vs. “control” categorical groups) were run, in order to identify the proteins that displayed a significantly altered expression level depending on those two different cultivation conditions. One hundred seventy-six statistically significant hits were identified on day 6 (Fig. 3a; also represented as a volcano plot in d), and 182 statistically significant hits were identified between “feed” and “control” on day 8 (shown in clusters in Fig. 3, panels b and c and as a volcano plot in d). The complete lists of these proteins, with corresponding log2 fold changes between tested groups (“feed” vs. “control,” day 6 and day 8), are viewable in the Supplementary Information, Table S1.

The differentially expressed protein profiles resulting from the categorical *t* tests between “control” and “feed” conditions on days 6 and 8 are also illustrated in Fig. 3, panels b-d. Profile plots of the log2 ratios for “feed” vs. “control” (panel b) are based on the hierarchical clustering map displayed in panel c. The latter show two distinct clusters: 86 proteins were significantly up-regulated, and 96 proteins were down-regulated, within the “feed” as compared to the “control” condition (on day 8). On the volcano plots (Fig. 3d), differentially expressed proteins are represented as a function of log2 ratio between F and C and statistical significance (x-axis: difference (*t* test fold change) vs. y-axis—log 10 *p* value). For the vast majority of these proteins, the regulation displayed the same trend on both day 6 and day 8—in other words, an up-regulated protein in “feed” on day 6 was also up-regulated on day 8, and vice versa. Out of 182 proteins with altered regulation reaching the *t* test significance threshold (permutation-based FDR < 0.05) on day 8, 88 were also observed to display a statistically significant regulation differential on day 6. All of these proteins were regulated in the same direction (i.e., up-regulated or down-regulated) at both of these time points for both the “feed” and “control” groups.

### Enriched gene annotations of regulated proteins

Those proteins that displayed statistically significant differences in their regulation between “feed” and “control” were thereafter subjected to a Fisher exact test with Benjamini–Hochberg FDR (BH-FDR) < 0.02. Proteins were annotated with molecular signature terms that were based on their gene names as identified per the Gene Ontology Cellular Component (GOCC), Reactome, Gene Set Enrichment Analysis (GSEA), Kyoto Encyclopedia of Genes and Genomes (KEGG), and CORUM molecular complexes database gene sets. The significance level of enriched annotations was assigned an adjusted *p* value of < 0.05 (after Benjamini–Hochberg false rate discovery correction). The initial Fisher exact test clustering data can be found in the Supplementary Information (Tables S3 and S4).

For day 8, three distinct major annotations containing a total of 42 non-duplicated genes were found: “extracellular matrix” (GO:0,031,012), “mitochondrial part” (GO:044,429), and the term “GSE11057_NAIVE_VS_CENT_MEMORY_CD4_TCELL_UP,” which comprised genes involved in metabolic processes, stress response, and cell cycle progression. Proteins that were initially categorized in more than one cluster were thereafter manually assigned to only one set (whichever had a higher enrichment factor and a lower *p* value). The identified proteins were thus curated and annotated according to their main biological function, based on sequence homology described in the scientific literature. These curated and annotated clusters are presented in Table 1.

### STRING analysis of proteins with enriched gene annotations

**Fig. 4:**
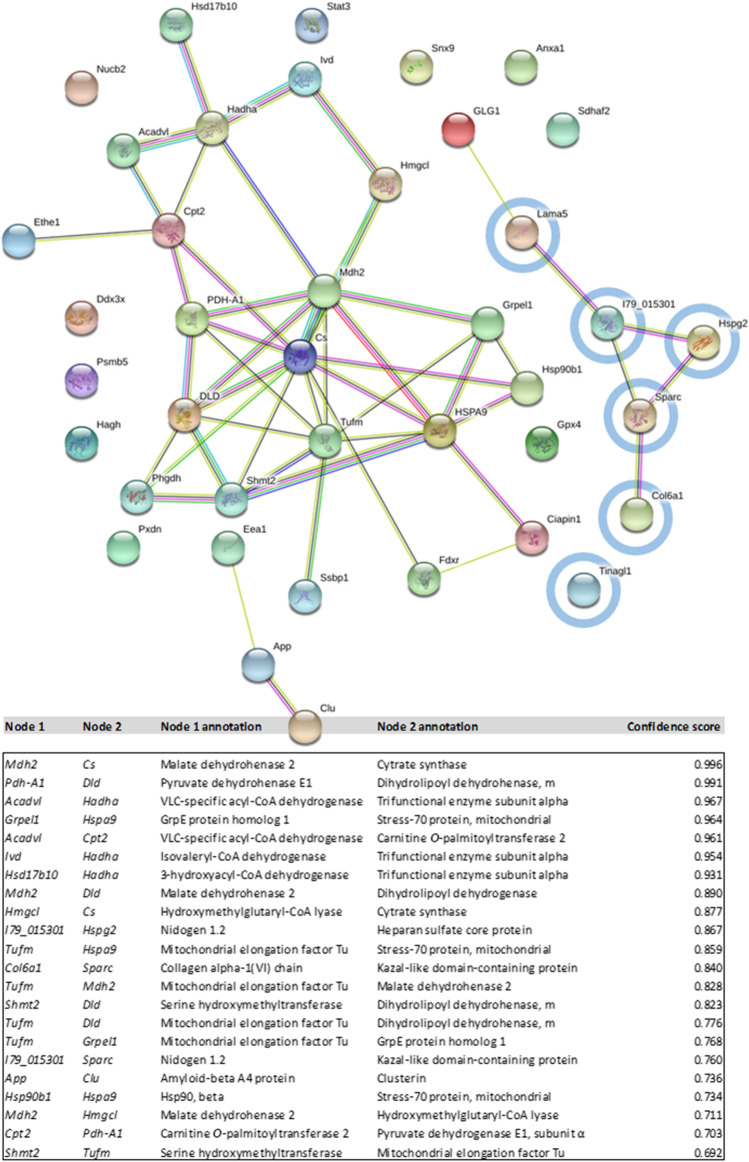
STRING visualization of the significantly regulated proteins with enriched annotations resulting from Fischer exact test BH-FDR < 0.02 listed in Table 1. The nodes (spheres) are the proteins and the connecting lines represent STRING interaction (according to STRING: red line, fusion evidence; green line, neighborhood evidence; blue line, co-occurrence evidence; purple line, experimental evidence; yellow line, textmining evidence; light blue line, database evidence; black line, co-expression evidence). Proteins annotated as extracellular cluster are circled in light blue. The table below represents confidence scores (the approximate probability that a predicted link exists in the same KEGG metabolic map) of the interaction nodes. The table below includes interactions with confidence scores ≥ 0.7 (“high confidence”) and ≥ 0.9 (“very high confidence”)

STRING visualizes interactions based either on similarity or existing evidence, and can be used as an additional tool to gain a better overview of protein scape alterations between samples. By using STRING, forty-one differentially expressed proteins containing enriched annotation terms based on their gene names (see Table 1; only one isoform, nidogen 1.1, was used) on day 8 were mapped. A resulting network image highlights the interconnectivity between these osmolality-impacted proteins (Fig. 4). Three clusters were identified via STRING within this set of proteins: (1) those connecting mitochondrial metabolism participants and parts of ROS-activated amelioration (twenty proteins); (2) those connecting basal membrane alterations (six proteins); and (3) those highlighting a recently identified interconnection between clusterin (Clu) and amyloid-beta A4 protein (Wojtas et al. 2017) (the smallest cluster, at just three proteins).

### “Top ten” lists of differentially regulated proteins

Based on the proteins that were significantly regulated on days 6 and 8 in “feed” vs. “control,” we also examined in greater detail the “top ten” proteins that displayed either statistically significant up-regulation or down-regulation (Table 2).

**Table 2:** The top ten significantly regulated proteins on day 6 and day 8 in “feed” vs. “control” conditions during the fed-batch cultivation of CHO DP-12 cells. The proteins in the top ten list are marked with “ +  + ” for day 6 or day 8, the proteins not in the top ten list but significantly regulated (permutation-based FDR < 0.05) on day 6 or day 8 are marked with “ + ”

	Gene name	Protein name	UniProt AC	Log2 fold change “feed”/ “control”		Top ten		Biological function
				**Day 8**	**Day 6**	**Day 8**	**Day 6**	
	Top ten up-regulated day 8 and day 6							
1	*Sprr1a*	Cornifin A	G3IIK9	4.92	3.91	+ +	+ +	Mitosis disruption
2	*Hmga1*	High-mobility group A1 proteins	G3IC63	3.65	3.19	+ +	+ +	Down-reg. cell proliferation
3	*Gpx1*	Glutathione peroxidase 1 (m)	G3H8G0	3.57	0.70	+ +	+	Cellular stress response
4	*I79_001876*	Ribosome-binding protein 1	G3GVX1	3.16	3.77	+ +	+ +	UPR in ER
5	*Nono*	Nono protein	A0A3L7H5A3	2.81	2.87	+ +	+ +	DSB repair factor
6	*H671_4g12516*	Septin 7	G3HTJ2	2.17	2.79	+ +	+ +	Filament-forming GTPase
7	*Ctsz*	Cathepsin X	Q9EPP7	2.00	1.98	+ +		Carboxypeptidase
8	*Ranbp2*	E3 SUMO-protein ligase RanBP2	G3HJ15	1.95	2.16	+ +	+ +	Stress protector
9	*Hspa5*	Heat Shock 70-kDa Protein 5	A0A3L7HCD3	1.73	1.04	+ +	+	Unfolded protein response
10	*Manf*	Mesencephalic astrocyte-derived neurotropic factor	G3H8A8	1.69	1.02	+ +	+	Stress response
11	*I79_021290*	Septin 11	G3IC99	1.28	2.10	+	+ +	Filament-forming GTPase
12	*CgPICR_005226*	Annexin	A0A3L7HVV8	1.38	2.09		+ +	Calcium ion binding
13	*H671_7g18400*	Septin 9	G3H3G9	0.74	2.07	+	+ +	Filament-forming GTPase
14	*I79_005051*	Nucleolar protein 56	G3H451	0.68	1.99	+	+ +	Ribosome biogenesis
	Top ten down-regulated day 8 and day 6							
1	*C1ra;C1rl*	Complement subcomponent C1r	G3GUR1	− 4.33	− 2.89	+ +	+ +	Ca^2+^ binding ser.-type protease
2	*Mt1*	Metallothionein	G3HIK0	− 3.82	− 3.32	+ +	+ +	Metal-ion detoxification
3	*Col6a1*	Collagen alpha-1(VI) chain	G3H8Y5	− 2.82	− 1.45	+ +	+ +	Part of extracellular matrix
4	*Tinagl1*	Tubulointerstitial nephritis antigen-like	G3H1W4	− 2.64	− 0.93	+ +		Laminin binding, cys.-type peptidase
5	*Notch2nl*	Notch homolog 2 N-terminal-like (preliminary data)	A0A3L7IFL8	− 2.30	− 0.99	+ +	+	Ca^2+^ binding, Notch2 binding
6	*Nedd4*	E3 ubiquitin protein ligase	A0A3L7IB07	− 2.08	− 1.46	+ +	+ +	Protein degradation
7	*Ubqln2*	Ubiquilin-2	G3HWU6	− 2.00	− 1.20	+ +	+	Protein degradation
8	*Hspg2*	Heparan sulfate proteoglycan core protein (preliminary)	A0A3L7I8L8	− 1.99	0.21	+ +		Basement membrane proteoglycane
9	*Glg1*	Golgi apparatus protein 1	G3I369	− 1.95	0.25	+ +		Down-reg. protein processing
10	*Ctla2a/Ctla2b*	cytotoxic T lymphocyte-associated protein 2 alpha/beta	G3IGW0	− 1.92	− 1.49	+ +	+ +	Down-reg. protein processing
11	*H671_6g15591*	Olfactory receptor 4P4-like protein	A0A061HXS4	− 0.65	− 2.03		+ +	RNA binding factor, transmembrane
12	*Ftsj3*	pre-rRNA processing protein FTSJ3	G3HCU9	− 0.51	− 1.79		+ +	rRNA binding methyltransferase
13	*Pbk*	Lymphokine-activated killer T-cell-originated protein kinase	G3HNI7	− 1.40	− 1.68		+ +	Mitotic cell cycle kinase
14	*Stat1*	Signal transducer and activator of transcription	G3I9F9	n/a	− 1.46		+ +	Centrosome doubling
15	*Dcps*	m7GpppX diphosphatase	G3HFJ1	− 0.03	− 1.44		+ +	mRNA degradation

In Table 2, the first part is dedicated to the top ten hits shared at both time points, followed by proteins in the top ten for day 8 and day 6 only. Mean log2 fold changes were calculated for LFQ-MS-based protein intensities in “feed” vs. “control” groups. The proteins included in this top ten list are indicated with “ +  + ,” while those excluded from this list—but which still displayed statistically significant regulation changes (permutation-based FDR < 0.05)—are marked with “ + .” Among the top ten up-regulated proteins in “feed,” six were common between days 6 and 8.

Similar to the up-regulation patterns observed on days 6 and 8, half of the top ten statistically significant down-regulated proteins were the same on both time points.

### Western blot analysis of the selected differentially expressed targets

We confirmed two differentially expressed proteins (Tinagl1 and septin 7) by a second, independent method. The label-free quantitative proteomics results for these targets were verified using western blot analysis (Fig. 2). According to MS results, septin 7 was significantly up-regulated in the feed-exposed group on both day 6 and day 8 (log2 fold change “feed” vs.”control” day8 + 2.17, day 6 + 2.79). Western blots of septin 7 coincide with the results of this MS analysis, and also suggest a clear up-regulation of the protein. A 50 kDa specific band was detected throughout all six samples (i.e., both “control” and “feed” on days 2, 6, and 8, respectively). The more intensive specific bands within the feed-exposed samples on day 6 and on day 8 (Fig. 2) also exhibited a narrowly spaced additional band, which we believe suggests the presence of either a septin 7 isoform or some alternatively modified protein.

Western blot analysis additionally confirmed the presence of a 52 kDa target band for Tinagl1. Although the antibody that was used did not show any exclusive specificity, we were able to detect the band at the correct height. An additional band at about 44 kDa was also detected by the antibody in CHO DP-12 samples and confirmed in lysates derived from human embryonic kidney cells (HEK) cells. Tinagl1 protein (UniProtID G3H1W4, *C. griseus*) has two *N*-linked glycosylation sites at positions Asn77 and Asn160. Presence of *N*-glycans influences significantly the migration of the protein in the gel and may explain the presence of several detectable bands (Unal et al. 2008) seen in our analysis. The intensities of both of these bands—i.e., at 52 kDa and 44 kDa—are in accordance with the down-regulation in “feed” at days 6 and 8 that was observed within the proteome data. Enhanced luminol-based chemiluminescent (ECL) substrate detected only one band at 250 kDa (Supplemental Fig. S1). As Tinagl1 binds the 70 kDa N-terminal segment and first type-III domain repeat of fibronectin (Li et al. 2007), a complex multimeric protein (Schwarzbauer and DeSimone 2011) together with laminins, nidogens, and collagen helps to form the extracellular matrix (ECM) of a CHO cell, and we assume that the detected band at 250 kDa can be attributed to a specific unresolved ECM protein complex.

## Discussion

### Enriched annotations of regulated proteins on day 8 reveal mitochondrial metabolism activation, basement membrane changes, and response to oxidative stress

**Fig. 5:**
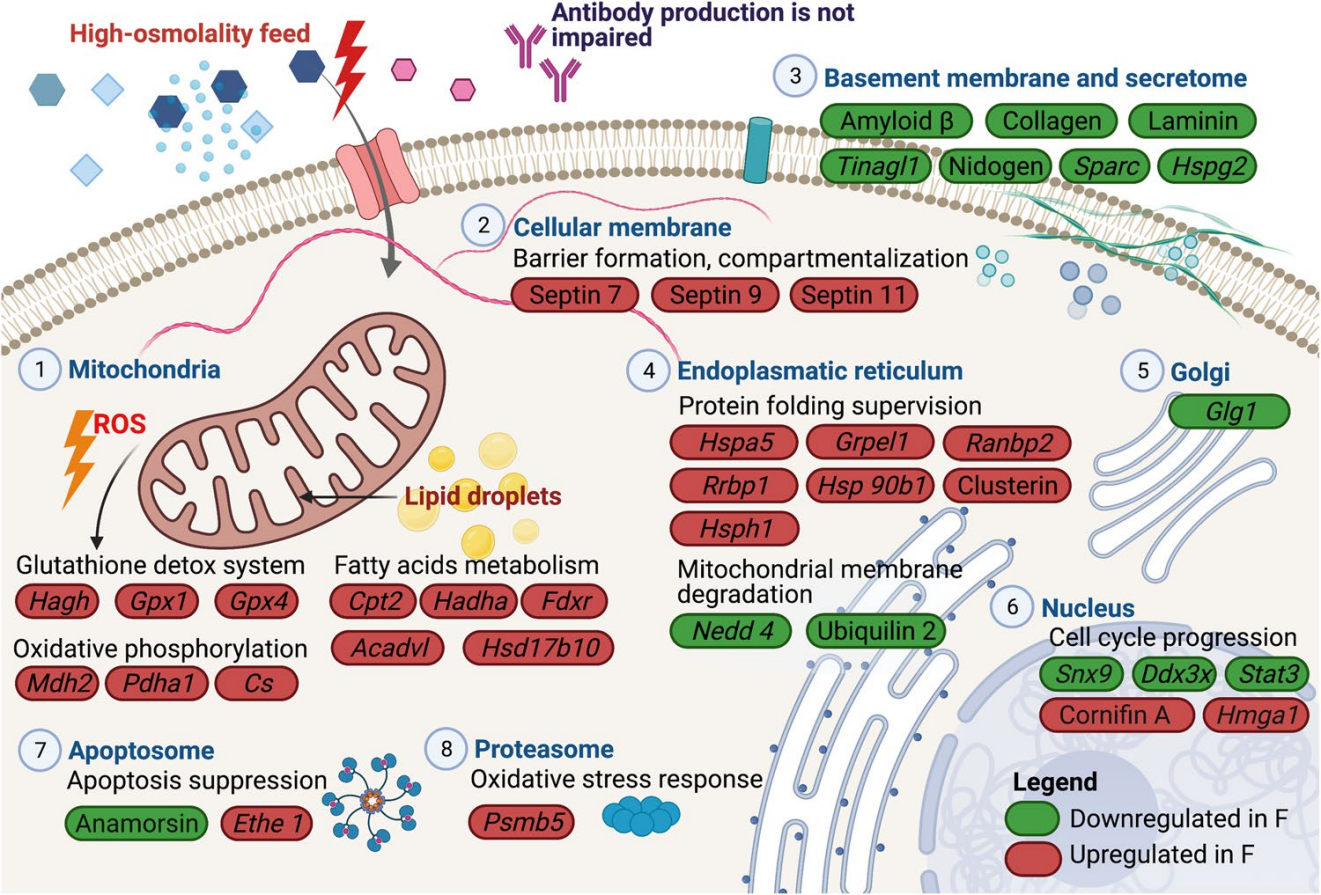
The major differentially expressed proteins on day 6 and 8 of the fed-batch cultivation between hyperosmolality-exposed CHO-DP12 cells (“feed”) and culture under physiological conditions (“control”). The proteins are arranged according to cellular structures they belong to. Green: proteins, significantly down-regulated in “feed” (Log2 fold change day 8 “feed” vs. day 8 “control” ≥ -0.7); red: proteins, significantly up-regulated in “feed” (Log2 fold change day 8 “feed” vs. day 8 “control” ≥  + 0.7). For full names of the proteins, please refer to Tables 1 and 2. Created with BioRender.com

The most significantly regulated proteins discussed in this work are presented graphically (according to their cellular components) in Fig. 5.

The gene ontology enrichment analysis results (Table 1) cluster labeled “extracellular matrix” encompasses the abundant membrane (App (amyloid-beta A4 protein), Sparc (kazal-like domain-containing protein), and Lama5 (laminin)), as well as mostly secreted proteins (Nid1.1 and Nid 1.2 (nidogen), Col6a1 (collagen, also in the top ten list), and Tinagl1 (tubulointerstitial nephritis antigen-like, also in the top ten list)). All of these proteins are components of the residual ECM of suspension-grown CHO DP-12 cells (Lu et al. 2011), and were less abundant on day 8 within the “feed” versus the “control” condition. Such coordinated down-regulation of Lama5, collagen, Nid1, and Tinagl1 might suggest a possible co-expression of these proteins in mammalian cells and has been reported previously (Clotet-Freixas et al. 2020). Western blot analysis also independently confirmed the presence and the down-regulation of Tinagl1 (Fig. 2a). Both ECM and secretome generation and remodeling are energetically expensive, but vitally inferior (Kol et al. 2020). Thus, such coordinated cutback on ECM protein expression in response to oversupplemented feed exposure may hint at cells’ striving to redirect energy to support more vital processes by cutting back on less prioritized activities.

The cluster labeled “genes involved in stress response” (Table 1) encompasses protein chaperones, peroxidases, and proteasome subunits that are active in protein degradation. Activation of oxidative stress response upon exposure to hyperosmotic media has been reported for CHO cells (Pan et al. 2019) and other cells (Li et al. 2017a, b; Xu et al. 2019). We surmise that the observed increase in numerous proteins involved in ROS sequestration in the cells that were exposed to the high-osmolality feed was a response to the activation of oxidative metabolism in mitochondria, and not vice versa.

Significant enrichment of proteins involved in mitochondrial regulation and function (Cluster IV in Table 1) provides strong evidence in support of our findings concerning mitochondrial activity, based on flow cytometry fluorescence measurements (Romanova et al. 2021), and ATP-pool enhancement under NaCl-induced high-osmolality, as previously reported (Pfizenmaier et al. 2015). It is also interesting to note that a pioneering proteome study of CHO under osmotic stress identified only two up-regulated proteins (pyruvate kinase and glyceraldehyde-3-phosphate dehydrogenase)—both of which are related to mitochondrial oxidative metabolism (Lee et al. 2003). All genes belonging to this cluster are up-regulated in the “feed” condition. The proteins coded by genes *Hadha*, *Hsd17b10*, *Cpt2*, and *Hmgcl* all regulate fatty acid β-oxidation and participate in lipid metabolism. Recent findings elucidate the clear connection between mitochondrial fatty acids metabolism, lipid droplets accumulation (previously reported for CHO under increased osmolality conditions (Pan et al. 2017), and cellular stress response (Jarc and Petan 2019).

Intracellular lipid droplets help to maintain membrane saturation and prevent peroxidation damage (Ackerman et al. 2018). They also serve as an energy storage buffer, supplying lipids for energy production and thus helping to enable the survival of stressed cells. Fatty acid oxidation in mitochondria increases NADPH levels in stressed cells (Jeon et al. 2012), which is in turn consumed for the regeneration of reduced glutathione (GSH), which serves as a substrate for glutathione peroxidase (Gpx4, included in cluster II (log2 fold change “feed” vs. “control” on day 8 + 1.04) and Gpx1 (log2 fold change “feed” vs. “control” day 8 + 3.56) in the top ten list)—the major antioxidant enzymes in glutathione-mediated elimination of H_2_O_2_.

The cluster of proteins associated with cell cycle progression encompasses proteins mediating proliferation arrest, as has been observed in all experiments with a hyperosmolality challenge. However, on day 8 of the fed-batch cultivation, cells in “control” used as a reference already exit the high-proliferation exponential growth phase; thus, no extensive list of differentially expressed proteins should be expected at this time point. The last cluster contains three genes that are involved in calcium-level maintenance (nucleobindin, an abundant Golgi-protein with high affinity to Ca^2+^ (Lin et al. 1999), (log2 fold change “feed” vs. “control” day 8 + 0.59)), as well as proteins that are involved in endosomal trafficking and L-serine biosynthesis. Ca^2+^ is a by-product of mitochondrial oxidative respiration and, in higher concentrations, is toxic to cells (Zorov et al. 2014). Nucleobindin binds and stores excessive calcium, which protects the cells from its damaging effects. Thus, the increased abundance of nucleobindin observed in “feed” cells might be connected with an increase in mitochondrial activity.

### STRING clustering highlights the interconnection of mitochondria-related proteins and basement membrane components

By feeding the proteins (Table 1) into the STRING database, we were also able to visualize the interactions between single members found to be enriched in the “feed” vs. “control” condition. As expected, STRING linked the members of citrate cycle Mdh2, Cs, PDH-A1, and DLD with the highest confidence scores (either ≥ 0.7 for “high confidence” or ≥ 0.9 for “very high confidence”) (Fig. 4). The intrinsic connection between mitochondrial fatty acid metabolism, oxidative phosphorylation, and neutralization of emerging ROS is also highlighted. In the second cluster, the members of the extracellular matrix cluster were mostly interconnected (Fig. 4, circled in light blue). Previous data from the relevant literature has suggested the influence of Sparc protein on collagen (Col6a) fibrils maturation (Rentz et al. 2007), and nidogen (I79_015301) is known to interconnect the laminin (Lama) and collagen networks with the assistance of perlecan (Hspg2) to help form a rigid basement membrane (Singh et al. 2012). Finally, the biological connection of the members of the last cluster encompassed only three proteins—amyloid-beta A4 protein (App), clusterin (Clu), and an early endosome antigen (Eea1) protein that is known predominantly from Alzheimer’s disease research (Wojtas et al. 2017, 2020; Zhao et al. 2021). The authors of the latter study found that overexpression of Clu (log2 fold change “feed” vs. “control” day 8 + 0.80) suppresses the formation of amyloid fibrils (log2 fold change “feed” vs. “control” day 8 − 1.88). The possible significance of such regulation in the context of CHO cells that are exposed to oversupplemented feed remains unclear.

### The top ten up-regulated proteins counteract oxidative stress, mediate cell cycle arrest, and increase membrane stability

Among the top ten up-regulated proteins in “feed,” six were common between days 6 and 8. The main findings discussed in this work are graphically summarized in Fig. 4. The two top hits—cornifin A and high-mobility group proteins A1 (HMGA1, formerly HMG-I/HMG-Y)—are both involved in cell cycle progression (Tesfaigzi et al. 2003) and mitogen-activated protein kinases (MAPKs)-mediated stress response (Schuldenfrei et al. 2011).

Cornifin A (log2 fold change “feed” vs. “control” day 8 + 4.92, day 6 + 3.91) belongs to the family of small proline-rich proteins (SPRR), which is expressed in CHO cells and is related to peptide cross-linking. Cornifin A becomes cross-linked with membrane proteins, and may therefore influence cell-envelope permeability (Marvin et al. 1992). It has been quantified in all biological replicates in both “feed” and “control,” and its homolog has also been identified in non-differentiating CHO cells (Tesfaigzi and Carlson 1996) and has been related to cells’ withdrawal from a proliferative state and the disruption of normal mitosis (Gibbs et al. 1993; Tesfaigzi and Carlson 1996; Tesfaigzi et al. 2003).

The high-mobility group A1 (HMGA1) proteins (HMGA1a and HMGA1b) (log2 fold change “feed” vs. “control” day 8 + 3.65, day 6 + 3.19) are members of the high-mobility group superfamily (HMG), which are non-histone nuclear proteins that participate in numerous biological processes including transcription, replication, cell cycle progress, DNA structure modification, and apoptosis (Fusco and Fedele 2007; Reeves 2000; Reeves and Beckerbauer 2001). The downstream transcriptional targets of HMGA1 are numerous, and include (by way of example) regulation of mitogen-activated protein kinases (MAPKs) MAPK11, MAPK12, MAPK13, and MAPK14 (Schuldenfrei et al. 2011)—all of which are major regulator molecules of the cell. They are rapidly activated by osmotic stress and other extracellular stress stimuli, such as UV-radiation and heat shock (for further information on this, see Zhou et al., (2016)). Recent research has revealed a synergistic regulation of signaling dynamics between MAPK and cyclin-dependent kinases (CDKs) (Repetto et al. 2018), and CDKs also seem to play an important role in cell size increase connected to an osmotic change in CHO cells (Pan et al. 2019). Although the precise function of HMGA1 overexpression in “feed” is unclear at this point, the authors speculate that it may display its function through these downstream effectors. The top ten up-regulated proteins were involved in stress and unfolded protein response (corresponding genes: *Gpx1*, *Rrbp1*, *Ranbp2*, *Hspa5*, and *Manf*) and DNA repair (*Nono*), and thus help to establish a balance between protein synthesis and degradation that promotes the survival of the cell.

Mitochondrial antioxidant enzyme glutathione peroxidase-1 (Gpx1) (log2 fold change “feed” vs. “control” day 8 + 3.57, day 6 + 0.69) and Gpx4 (discussed in the previous section) are abundant mammalian selenoenzymes that play a prominent role in the cellular response to reactive oxygen species (ROS)-induced oxidative stress (Chevallier et al. 2020). The term “ROS” includes oxygen free radicals—such as superoxide anion radical (O_2_^−^) and hydroxyl radical (OH)—as well as non-oxidant radicals such as hydrogen peroxide (H_2_O_2_) and singlet oxygen (^1^O_2_). Despite several antioxidant defenses, the mitochondrion appears to be the main intracellular source of these oxidants (Turrens 2003). In particular, Gpx1 couples oxidation of glutathione, a small three-residue (γ-L-glutamyl-L-cysteinyl glycine) peptide serving as an electron donor with H_2_O_2_ detoxification (Espinosa-Diez et al. 2015; Ighodaro and Akinloye 2018). Its protective role has been shown for knockout mice in coping with oxidative injury and death mediated by reactive oxygen species (Esposito et al. 2000), as well as for CHO cells exposed to oxidative stress (Aykin-Burns and Ercal 2006). Its up-regulation in the “feed” suggests that Gpx1 is also strongly involved in mitigating oversupplemented feed-induced stress.

Ribosome-binding protein 1 (RRBP1, log2 fold change “feed” vs. “control” day 8 + 3.16, day 6 + 3.77) is localized on the rough endoplasmic reticulum (ER) and is part of unfolded protein response (UPR) that is induced during cellular stress (Gao et al. 2016). RRBP1 alleviates ER stress, and thus helps to facilitate cell survival (Tsai et al. 2013).

SUMO E3 ligase Ran-binding protein 2 (RanBP2, log2 fold change “feed” vs. “control” day 8 + 1.95, day 6 + 2.16) is a large protein with multiple functions. It is known to directly modulate responses to phototoxicity, infectious agents, and carcinogens (Cho et al. 2010), and also to suppress apoptosis during oxidative stress (Cho et al. 2012). Specifically, RanBP2 co-localizes with and modulates the activity of mitochondrial proteins (Patil et al. 2019). It is noteworthy that a very high up-regulation of the ortholog gene *Ranbp3l* was recently reported in a transcriptome study of suspension-adapted CHO cells under elevated osmolality (Pan et al. 2019).

Mesencephalic astrocyte-derived neurotrophic factor (Manf, log2 fold change “feed” vs. “control” day 8 + 1.69, day 6 + 1.02), as well as Bip/Hspa5 protein chaperons (Hamman et al. 1998), is activated in response to mild hypothermia in CHO cells (Bedoya-López et al. 2016), and also seem to play a role in response to glucose-induced hyperosmolality. Hspa5 is a master chaperone that assists in the translocation, folding, and stabilization of nascent protein chains.

An RNA- and DNA-binding non-POU domain-containing octamer-binding (NONO) protein is a candidate DNA double-strand break (DSB) repair factor, showing end-joining stimulatory activity in biochemical protein screening (Li et al. 2017a, b).

Aside from oxidative stress amelioration, we expected to find that some specific adaptation proteins counteracted the degradation of a CHO cell’s spherical shape. Septins are a family of filament-forming GTPases that interact with both the F-actin protein and the microtubule skeleton of the cell. Three members of this family are found in our top ten list: septin 7 (log2 fold change “feed” vs. “control” day 8 + 2.17, day 6 + 2.79), septin 11 (log2 fold change “feed” vs. “control” day 8 + 1.28, day 6 + 2.10), and septin 9 (log2 fold change “feed” vs. “control” day 8 + 0.74, day 6 + 2.07). Exemplary for this protein family, the presence and up-regulation of septin 7 have been also confirmed by western blot analysis (Fig. 2). The membrane-binding ability of septins, which limits the lateral diffusion of membrane proteins (Caudron and Barral 2009; Fung et al. 2014; Gilden et al. 2012), may alter the permeability and rigidity of the cellular membrane during osmotic pressure elevation. Although there is no reported experimental data for CHO cells on this point, osmotic change experiments on septin-coated vesicles do indeed suggest an increase in rigidity and shape stability under hyperosmotic conditions when compared to uncoated vesicles (Beber 2018). The fact that members of this septin family rank among the top ten most up-regulated expression proteins in the “feed” group hints that their role in increasing membrane stability and thus facilitating cellular homeostasis maintenance plays a crucial role in supporting cell survival under osmotic pressure.

Although limiting the number of proteins examined in more detail to a top ten is somewhat arbitrary (in that it does not necessarily provide a full and comprehensive picture of the entire cellular regulation process), we believe that it does offer valuable insight into the predominant changes that were observed between the two conditions that we studied. To summarize the foregoing, a majority of the top ten up-regulated proteins in “feed” on both day 6 and day 8 were proteins that are known to be involved in processes such as oxidative stress amelioration, protein aggregation mediation, and propagating cell cycle arrest. At last, membrane rigidity and permeability appear to be increased via the accumulation of septins and cornifin A.

### The top ten down-regulated proteins are involved in protein degradation, RNA processing, and cell cycle arrest

The majority of the top ten down-regulated proteins identified the in “feed” group are involved in proteolysis (corresponding genes *C1ra/C1rl*, *Nedd4*, *Ubqln2*, *Ctla2a/Ctla2b*) and managing alterations in RNA processing (*H671_6g15591*, *Ftsj3*, *Dcps*). Taken together, this seems to indicate that CHO cells exposed to oversupplemented feed are not only engaged in protein biosynthesis (as previously reported in Pan et al. 2017) but also strive to preserve already-expressed proteins from degradation. The input of these processes would also help to explain the accumulation of cellular dry mass that has been observed in CHO cells exposed to osmotic stress (Pan et al. 2017). In further support of this hypothesis, it is worth noting that prior research has demonstrated that proteasomal degradation decreases at higher oxidant levels (Breusing and Grune 2008), and that the down-regulation of those proteins involved in protein degradation that were included within the top ten list became more pronounced from day 6 to day 8.

Two of the down-regulated proteins in the top ten—ubiquilin Ubqln2 (log2 fold change “feed” vs. “control” day 8 − 2.00, day 6 − 1.20) and ubiquitin protein ligase E3Nedd4 (log2 fold change “feed” vs. “control” day 8 − 2.08, day 6 − 1.46)—also seem to be related to the process of mitochondrial up-regulation. Ubiquilins are chaperones that protect transmembrane proteins, and they usually exhibit long hydrophobic regions from aggregation in the polar cytosol (Zhang et al. 2014). Ubqln1 and 2 interact specifically with mitochondrial transmembrane proteins and degrade them upon failure to be inserted into their target. To facilitate ubiquitination, ubiquilins recruit E3 ligase (coded by Nedd4) (Itakura et al. 2016). Thus, concomitant down-regulation of these two components in the “feed” group might indicate a cutback of mitochondrial membrane degradation—potentially leading to an increase of mitochondrial fluorescence, due to an increased abundance of the membrane proteins which accumulate fluorescent dye (Romanova et al. 2021).

Down-regulation of Pbk-coded lymphokine-activated killer T-cell-originated protein kinase (TOPK, log2 fold change “feed” vs. “control” day 8 − 1.40, day 6 − 1.68) and Stat1 (log2 fold change “feed” vs. “control” day 8 not quantified, day 6 − 1.46), coding for signal transducer and activator of transcription (quantified only for the day eight), seems to be related to CHO cell cycle arrest. TOPK expression peaks as proliferating cells enter mitosis (Herbert et al. 2018) and its knockout induces G1-phase cell cycle arrest in cancer cells (Zhao et al. 2020). Stat signaling is involved in numerous processes in the cell, including a centrosome doubling during cell cycle progression in CHO cells (Metge et al. 2004), and is obviously not required when the cells do not proliferate, which may explain the down-regulation of Stat protein in the “feed.”

To summarize the foregoing, then the known effects of the top ten down-regulated proteins in the “feed” group collectively hint at a decrease in protein degradation, alterations in RNA processing, and the exit of CHO cells from their normal proliferative pattern.

Using the LFQ approach, combined with stringent statistical analysis and comprehensive data mining and evaluation, it was possible for the authors to identify some of the most significant proteome changes that appear to be caused by exposure of CHO DP-12 cells to highly concentrated feed (where D-glucose functioned as the main component causing the osmolality increase). In the course of this analysis, we were able to quantify a total of 1798 proteins—of which 176 were significantly regulated on day 6, and 182 were significantly regulated on day 8—observed in the “feed” culture as compared to the “control” culture at the same time points. The presence and regulation of two target proteins (septin 7 and Tinagl1) were additionally confirmed via western blot analysis. Specifically, this analysis revealed a significant up-regulation of proteins that are known to mediate oxidative stress, protein response, and protein degradation processes. We also detected a down-regulation of multimodal regulator molecules (Stat3 and Stat1), which are known to be involved in cell cycle progression, as well as an up-regulation of cornifin A and Ddx3x, which are known to mediate cell cycle arrest. Together, these regulated proteins would all seem to be involved in the proliferation termination that was observed in the “feed” group during the fed-batch cultivation. The up-regulation of oxidative stress response proteins, present both in our top ten lists (discussed above) and in the gene ontology enrichment clustering, is likely caused by an elevated level of mitochondria-derived ROS. Accordingly, we believe that the higher expression of oxidative stress response proteins that we observed is likely linked to activation of mitochondrial oxidative phosphorylation and lipid oxidation that was detected via a molecular annotation enrichment analysis on the proteome level in our setup. It is known that activation of both oxidative phosphorylation and lipid oxidation yields an excess of ATP in response to NaCl-caused hyperosmolality in CHO culture (Pfizenmaier et al. 2016). The excess of energy produced by mitochondria seems to be used for the synthesis of proteins that prevent protein aggregation and alleviate oxidative stress, as well as those which play a role in maintaining cellular membrane rigidity and permeability, such as septins. These activated high-osmolality-dependent processes are accompanied by the down-regulation of proteases which indicate a reduction in protein degradation. Continued biomass synthesis, paired with protein degradation suppression and proliferation stop, thus results in considerable cellular mass and volume accumulation. Though our proteome data is entirely in concordance with the relevant previously reported literature, the granular evaluation and identification of the specific biological function of each target identified in this study must ultimately be validated only by more targeted approaches undertaken in future work—such as interaction analysis, knock-out, silencing-mRNA, or similar experiments. Nonetheless, we believe that the findings reported in this paper may be useful for developing a more informed and precise feed design that promotes greater cultivation process optimization.

## Supplementary Information

Below is the link to the electronic supplementary material.

Supplementary file1 (PDF 619 KB)

Supplementary file2 (XLSX 2017 KB)

## Data Availability

Data are available via ProteomeXchange, with identifier PXD027443.
