# Supplementary material for: Hyperosmolality in CHO cell culture: effects on the proteome
Source: Appl Microbiol Biotechnol. 2022 Mar 21;106(7):2569–86. doi: 10.1007/s00253-022-11861-x (PMC8990941; doi:10.1007/s00253-022-11861-x)
Supplement: Supplementary file 1 — Supplementary file1 (PDF 619 KB) [file 253_2022_11861_MOESM1_ESM.pdf]

Applied Microbiology and Biotechnology

Supplementary figure for a research paper entitled "Hyperosmolality in CHO Culture: Effects on the Proteome"

Nadiya Romanova[1],Louise Schelletter[1],Raimund Hoffrogge[1], Thomas Noll[1*]

[1]: Cell culture technology, Technical Faculty, Bielefeld University, Bielefeld, Germany

*Correspondence concerning this article should be addressed to Thomas Noll, Bielefeld University, Universitätsstraße 25, 33615 Bielefeld Germany. E-mail: Thomas.Noll@uni-bielefeld.de; Tel. +49 521 106-6319; Fax +49 521 106-6318.

Number of Supplementary Material pages (including this title page): 2

Fig. S1 Western blot analysis using ECL substrate of oversupplemented feed-exposed (F) and control (K) whole protein lysates of the fed-batch cultivation of CHO-DP12 sampled on days 2, 6 and 8 (D2, D6 and D8 in the figure). Primary polyclonal antibody against Tinagl1, detecting a specific band at about 230 kDa probably consistent of multiple unresolved proteins of ECM.

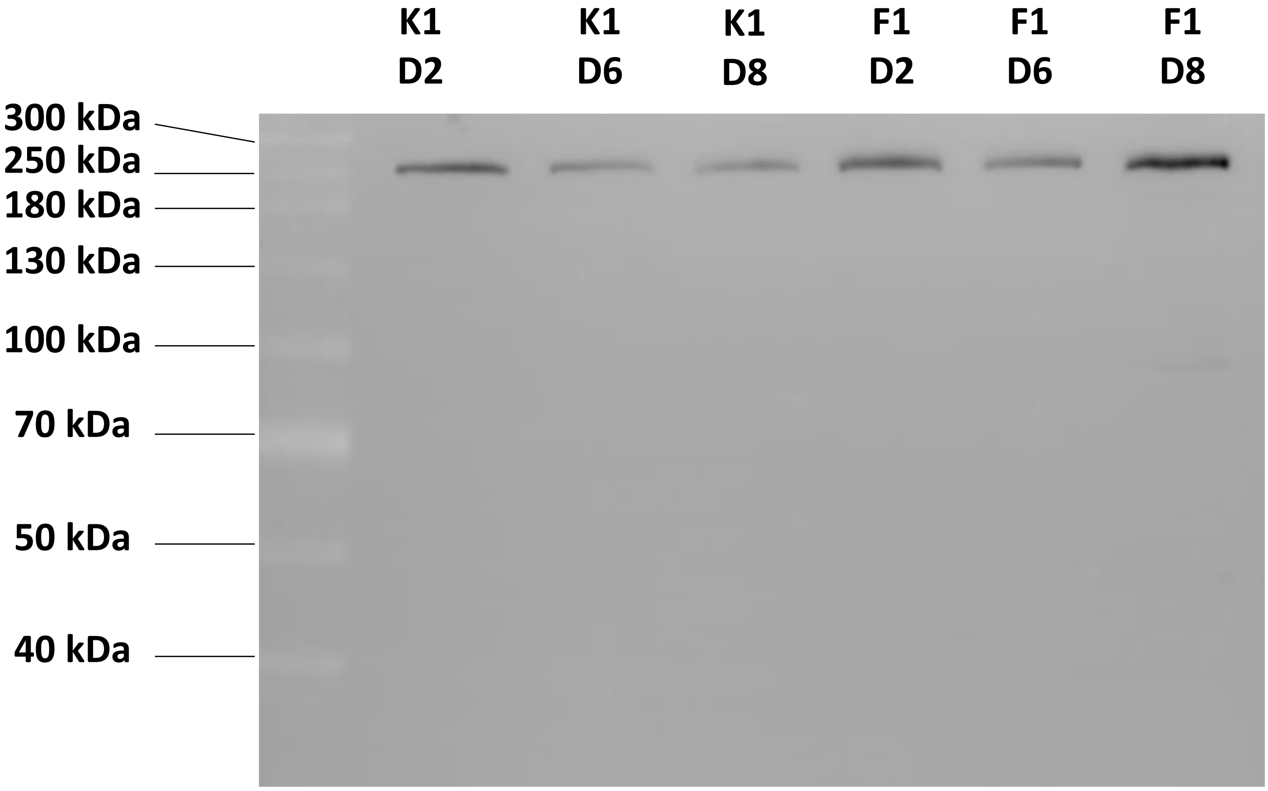
K1
D2
K1
D6
K1
D8
F1
D2
F1
D6
F1
D8
300 kDa
250 kDa
180 kDa
130 kDa
100 kDa
70 kDa
50 kDa
40 kDa
